# Influence of varying estrogen levels on trigeminal CGRP release in healthy women

**DOI:** 10.1186/1129-2377-14-S1-P123

**Published:** 2013-02-21

**Authors:** K Ibrahimi, AHJ Danser, CM Villalón, AH van den Meiracker, A MaassenVanDenBrink

**Affiliations:** 1 Erasmus MC, Netherlands; 2 Cinvestav Coapa, Mexico

## 

Migraine is 2-3 times more prevalent in women than in men, with frequent perimenstrual attacks. TRPV1 receptors on sensory nerve endings of the trigeminal track are important in mediating migraine attacks by releasing the vasodilator calcitonin gene-related peptide (CGRP). Variation in estrogen levels during the menstrual cycle may have an influence on the sensitivity of the TRPV1 receptor or on the amount of CGRP in perivascular nerve terminals and hence on CGRP release. Capsaicin, the active ingredient of hot chili peppers, stimulates the TRPV1 receptor and causes CGRP-dependent vasodilatation [1]. We set up a model to study trigeminal CGRP release in humans. We compared the vasodilator effects of capsaicin application and electrical stimulation on the forehead skin. Healthy women, not using hormonal contraceptives (age: 18-45, n=14), were studied with a Laser Doppler Imager on day 19-21 of their menstrual cycle and on day 1-2 of their menstruation. A 0.2 mM and a 20 mM capsaicin solution were applied to the skin. In addition, iontophoresis of saline was performed as a TRPV1-independent stimulus. Increases in dermal blood-flow (DBF) were measured. Blood samples were collected to measure estrogen levels. We measured higher DBF responses to application of 0.2 mM capsaicin (Max:226±34 a.u.) and 20 mM capsaicin (Max:507±39 a.u.) during day 1-2 (low estrogen levels: 15±2 pg/ml) of the menstruation, than during day 19-21 (high estrogen levels: 67±9 pg/ml) of the menstrual cycle (Max:176±34 a.u. and 432±33 a.u. for 0.2 mM and 20 mM, respectively, P<0.05). There was no difference in DBF responses to electrical stimulation of the forehead skin, suggesting that the observed changes are related to the sensitivity of the TRPV1 receptor. Our results indicate an influence of variation in estrogen levels on trigeminal CGRP release, with the highest reactivity observed around the menstruation when estrogen levels are low. This mechanism may, at least partly, explain the high incidence of migraine attacks during the perimenstrual period.
